# Humanising nanotoxicology: replacement of animal-derived products in the application of integrated approaches to testing and assessment of nanomaterial inhalation hazard

**DOI:** 10.3389/fbioe.2025.1526808

**Published:** 2025-02-12

**Authors:** Roma Fraser, Keira Campbell, Pawel Pokorski, Eve MacKinnon, Katie McAllister, Karla B. Neves, Fiona Murphy

**Affiliations:** ^1^ Strathclyde Institute of Pharmacy and Biomedical Sciences, University of Strathclyde, Glasgow, United Kingdom; ^2^ Institute of Biological Chemistry, Biophysics and Bioengineering, Heriot Watt University, Edinburgh, United Kingdom

**Keywords:** 3Rs, human platelet lysate, *in vitro*, cell culture, new approach method (NAM)

## Abstract

Over the past decade, the development of nanomaterials (NMs) has surged, highlighting their potential benefits across multiple industries. However, concerns regarding human and environmental exposure remain significant. Traditional *in vivo* models for safety assessments are increasingly viewed as unfeasible and unethical due to the diverse forms and biological effects of NMs. This has prompted the design of Novel Approach Methods (NAMs) to streamline risk assessment and predict human hazards without relying on animal testing. A critical aspect of advancing NAMs is the urgent need to replace animal-derived products in assay protocols. Incorporating human or synthetic alternatives can significantly reduce the ethical burden of animal use while enhancing the relevance of toxicity testing. This study evaluates the impact of removing animal-derived products from standard acellular and *in vitro* assays recommended in a published Integrated Approaches to Testing and Assessment (IATA) for inhaled NMs. We specifically assessed the effects of replacing fetal bovine serum with human platelet lysate in acellular reactivity tests and *in vitro* toxicity testing using a panel of well-characterized NMs. Significant differences in acellular NM reactivity and dramatic changes in A549 cell growth rates and responses to NMs were observed under different media conditions. Our findings demonstrate that variations in experimental setup can fundamentally impact NM hazard assessment, influencing the interpretation of results within specific assays and across tiered testing strategies. Further investigation is needed to support a shift toward more ethical toxicity testing that does not rely on animal-derived materials.

## 1 Introduction

Over the last decade there has been a notable rise in nanomaterial (NM) development and production for a vast array of different applications. While the potential benefits of NMs for a wide range of industries are clear, concerns over the impact of human and environmental exposure remain. Since NMs can exist in many diverse forms, each of which may cause different biological effects, reliance on traditional *in vivo* models for safety assessment is not feasible, sustainable or ethical, considering the volume of materials that need to be tested (Burden et al., 2021). This has stimulated efforts to design alternative strategies to streamline the risk assessment process and Novel Approach Methods (NAMs) to predict human hazard without the need for animal testing (Stucki et al., 2022). Many of these NAMs are adapted from cell culture protocols that historically involve the use of animal-derived products therefore, to fully humanise the hazard assessment process it is pertinent to incorporate 3Rs principles into the design of NAMs and novel testing frameworks at this early stage of development. The central focus of this study is to assess the impact of removing animal-derived products from standard acellular and *in vitro* assays in order to reduce ethical concerns and increase the human relevance of toxicity testing.

Integrated Approaches to Testing and Assessment (IATAs) are a tool which can be utilised to streamline the hazard assessment process (Sakuratani et al., 2018). Structured as decision trees, IATAs enable the user to efficiently identify the key information required to support hazard assessment and risk decision making with regards to NM(s) under investigation (Stone et al., 2020). IATAs, delineated by the primary routes of NM exposure, have successfully been used as the basis of similarity assessment to support grouping of NMs which pose a comparable inhalation, ingestion, or dermal hazard (Di Cristo et al., 2022; Braakhuis et al., 2021; Murphy et al., 2021) and incorporated into a risk decision making framework for the injection of nanobiomedicines (Powell et al., 2022). Recognizing the need to reduce the burden of animal testing in toxicology, the IATAs follow a tiered structure and prioritize data generation utilizing *in silico* and *in vitro* models to categorize hazard before progressing to *in vivo* systems in limited, high priority cases (Stone et al., 2020).

To meet the necessary demand for improved *in vitro* models required to build confidence in the IATA outcome, a concerted effort has been made to develop NAMs which prove effective for predicting hazard (Doak et al., 2022). NAMs are generally defined as toxicological methods that serve as (replacement, reduction or refinement) alternatives to animal testing (e.g., *in silico*, *in chemico* and *in vitro* methods) (Burden et al., 2021). The shift to wider adoption of NAMs will have a seismic impact on the reduction of animal numbers used directly in toxicology studies, however the use of animals to generate reagents and supplements currently required in the standard operating protocols (SOPs) for many NAMs remains as a significant burden. The replacement of animal-derived products incorporated into the NAMs protocols has received less attention to date but as NAMs progress towards validation it is timely to consider how to optimise the NAM underdevelopment without the need for animal-derived products.

The removal of animal-derived products is likely to have biological, safety as well as ethical benefits, improving assay performance and reproducibility. Species-species differences in animal-derived products may have a significant impact on the performance of NAMs due to species-specific interactions between cells and proteins from different animal sources. For example, serum transferrin functions in transport and delivery of iron to cells (Aisen and Listowsky, 1980). Penhallow et al., showed a difference in the ability of human-derived cells to utilise iron from transferrins (Tf) isolated from the sera of human, equine or bovine sources which had an on impact cell growth (Penhallow et al., 1986). Enhanced assay specificity and sensitivity may be an additional benefit as human or synthetic-based reagents eliminate the potential for cross-reactivity or interference associated with animal-derived components within an assay (Liu et al., 2023). Reducing the potential for xenobiotic immunological responses is particularly relevant for to the toxic outcome is understood allowing structure-activity relationships between NM characteristics and toxic outcomes to be recognised.

The overall aim of this paper is to assess the impact of replacing animal-derived reagents with human-based alternatives when designing IATA and conducting tiered testing, in response to the current drive towards adopting animal-free approaches in toxicology.

A published IATA for inhalation exposure of NM was selected as an exemplar testing strategy for the hazard assessment of NM as inhalation is the primary route of concern (Braakhuis et al., 2021). The initial objective of this project was to conduct an audit of the assays and methods recommended within the IATA to identify where animal-derived products were included, whether these were necessary or, if appropriate, human-derived, or synthetic alternatives were available. As a case study, the impact of replacing animal-derived products was evaluated for both acellular assays and simple Tier 1 *in vitro* toxicity testing using A459 alveolar epithelial cells. A panel of well-characterised high and low toxicity NMs, were selected to assess the suitability of the animal-free approach to NM hazard assessment leading to the potential for an alternative ‘humanised’ IATA which minimises or avoids the use of animal-derived products.

## 2 Methods

### 2.1 NM suspensions

The panel of case study NMs (CuO, ZnO, CeO_2_, BaSO_4_, obtained from the EC Joint Research Centre Nanomaterial repository, Ispra, Italy) were selected based on an abundance of existing toxicity data from *in vitro* and *in vivo* models [7]. NMs were weighed into glass vials and diluted to 1 mg/mL concentrations using PBS supplemented with 2% FBS (Gibco) or 2% Human Platelet Lysate (HPL) (Stemcell Technologies). Before use, particles were dispersed by probe sonication for 30 s at 40% power. PBS supplemented with 2% FBS or 2% HPL were selected as NM dispersants to align with the recommended standard operating protocol for the acellular DCFH_2_-DA assay (Boyles et al., 2022).

### 2.2 Size and agglomeration status of NM panel

Dynamic light scattering (DLS) was measured using Malvern Zetasizer Nano ZS (Malvern Industries, Malvern, UK) to determine the hydrodynamic diameter and polydispersity index of each case study NM when suspended in PBS supplemented with 2% FBS or 2% HPL and dispersed by sonication directly prior to measurement.

### 2.3 DCFH_2_-DA assay

For each case study NM, prepared in PBS supplemented with 2% FBS or 2% HPL, the acellular generation of ROS was detected by using an oxidant-sensitive fluorescent probe, DCFH_2_-DA, following the standard operating protocol described in literature (Boyles et al., 2022). Fluorescent readings were detected using the GloMax^®^ Explorer microplate reader at 485/530 ex/em nm after 90 min incubation and were normalised to a standard curve of fluorescein fluorescence and expressed as Fluorescein Equivalent (μM). PBS supplemented with FBS or HPL included as the relevant vehicle control.

### 2.4 Ferric reduction ability of serum assay (FRAS)

The panel of NMs was used for assessment of the total antioxidant depletion level in commercially available human serum (H4522, Sigma-Aldrich). After a 3-h incubation of serum with the NMs at 37°C, the NM-mediated depletion of antioxidant capacity was quantified by colorimetric detection of Fe^2+^-2,4,6-tri (2-pyridyl)-s-triazine complexes according to the protocol reported in Gandon et al. (2017).

### 2.5 A549 cell culture

The cell line presents in this study were obtained from ATCC (A549 CCL-185). A549 human alveolar epithelial cells were continuously cultured in T75 cell culture flasks at 37°C with 5% CO_2_ and maintained in RPMI 1640 medium, supplemented with either 5% (v/v) HPL (Stemcell Technologies), 1% L-Glutamine (Gibco) and 1% (v/v) penicillin/streptomycin (Gibco) or 10% (v/v) FBS (Gibco) with 1% L-Glutamine (Gibco) and 1% (v/v) penicillin/streptomycin. Due to the higher protein content of HPL (56 mg/mL) compared to FBS (38 mg/mL) (according to respective product datasheets), a lower concentration of HPL was tested to maintain a more consistent total protein content across all tested supplements. The A549 cells, originally cultured in FBS, were adapted to HPL over several passages (minimum 3) prior to assessment and maintained continuously in the respective media over the course of growth curve analysis. The culture medium was renewed every 2–3 days for both conditions, and cells were subcultured upon reaching approximately 70% confluence following disruption with 1X TrypLE™ Express Enzyme (Gibco), an animal-free recombinant Trypsin-Like Enzyme.

### 2.6 A549 growth rate analysis

Growth rates were quantified by counting A549 cells cultured continuously in either FBS- or HPL-supplemented medium at days 0, 1, 2 and 3 using a hemocytometer, after disruption with 1X TrypLE™ Express Enzyme (Gibco), an animal-free recombinant Trypsin-Like Enzyme.

### 2.7 Cell morphology using light and fluorescent imaging

Morphological changes between A549 cells cultured in medium supplemented with FBS or HPL were visualised by light microscopy using the EVOS™ XL Core Imaging System. For immunofluorescent staining cells were seeded onto glass coverslips for 24 h and fixed with 4% paraformaldehyde (PFA) for 15 min before staining with Fluorescein Phalloidin (F432, Thermo Scientific) and mounted using ProLong™ Diamond Antifade Mountant with DAPI (P36966, Thermo Scientific). Slides were imaged using fluorescent microscopy at ×10 and ×40 magnifications.

### 2.8 Western blotting

A549 cells, cultured in FBS- or HPL-supplemented medium, were lysed using RIPA buffer (10 mM Tris-HCl, 1 mM EDTA, 0.5 mM EGTA, 1% v/v Triton X-100, 0.1% w/v SDS, 140 mM NaCl, diluted with dH_2_O, pH 8.0). Protein levels were quantified using a BCA assay (23225, Thermo Scientific). Equal amounts of protein samples were loaded per well and separated by sodium dodecyl sulfate-polyacrylamide gel electrophoresis (SDS-PAGE). Samples were electro-transferred to nitrocellulose membranes. The membranes were blocked with 5% v/w non-fat dry milk then incubated at 4°C overnight with primary antibodies to detect vimentin (SC-6260, Santa Cruz Biotechnology) and Glyceraldehyde 3-phosphate dehydrogenase (GAPDH), used as the loading control. The membranes were then incubated with secondary antibodies. Protein bands were visualised using LI-COR Odyssey^®^ 9120 Gel Imaging System (Biosciences).

### 2.9 Alamar blue

The Alamar Blue (AB) cell viability assay was used to measure cell viability of A549 cells in either HPL or FBS supplemented medium following exposure to the panel of case study NMs and relative to 0.1% v/v Triton X-100, the negative control (100% cell death). Confluent cells were trypsinised and counted, as detailed previously. Cells were seeded at an initial density of 2.7 × 10^5^ cells/well in 12-well plates, using medium supplemented with HPL or FBS, then incubated overnight. Cells were treated with six different concentrations of each test NM (5, 10, 20, 39, 78 μg/cm^2^) suspended in FBS- or HPL-supplemented media with the corresponding media included as the vehicle control. Treatments were incubated for 5- or 24-h. At the end of the treatment, medium was removed, and AB reagent (2% resazurin in PBS) was added to each well and incubated at 37°C for 30 min. The supernatant from each well was plated in triplicate on a 96-well plate then fluorescence detected using the GloMax^®^ Explorer microplate reader at ex/em 520 nm/580–640 nm.

### 2.10 Wound healing assay

Cells were seeded at a density of 2.7 × 10^5^ cells/well in 12-well plates, using medium supplemented with HPL or FBS, and incubated overnight at 37°C. A scratch was made along the centre of the well using a sterile p200 pipette tip. Wells were imaged by light microscopy using the EVOS™ XL Core Imaging System at time 0 and after 24 and 48 h. ImageJ was used to measure the gap width in each media conditions at 0-, 24-, or 48-h timepoints.

### 2.11 Fluorescent bead uptake

Cells were seeded at a density of 0.5 × 10^5^ cells/well in a 24-well plate and incubated with Fluoresbrite^®^ YG Carboxylate Microspheres 200 nm beads (1 × 10^4^ - 1 × 10^11^ beads/well) for 5 h. Supernatants were removed and cells washed twice with PBS. Fluorescence of wells were then measured on the GloMax^®^ Explorer microplate reader at ex/em 414 nm/488 nm.

### 2.12 Statistical analysis

The data are presented as mean ± SEM of at least three independent samples or experiments, and was analysed using GraphPad Prism 9, version 9.4.1 (GraphPad Software Inc., San Diego, California, United States). When two groups were compared, Student’s t-test was to compare mean values between groups. When applicable, ordinary one-way ANOVA using multiple comparisons was used to elucidate statistical differences between multiple groups. Statistical significance was set at p < 0.05.

## 3 Results

### 3.1 IATA audit

**FIGURE 1:**
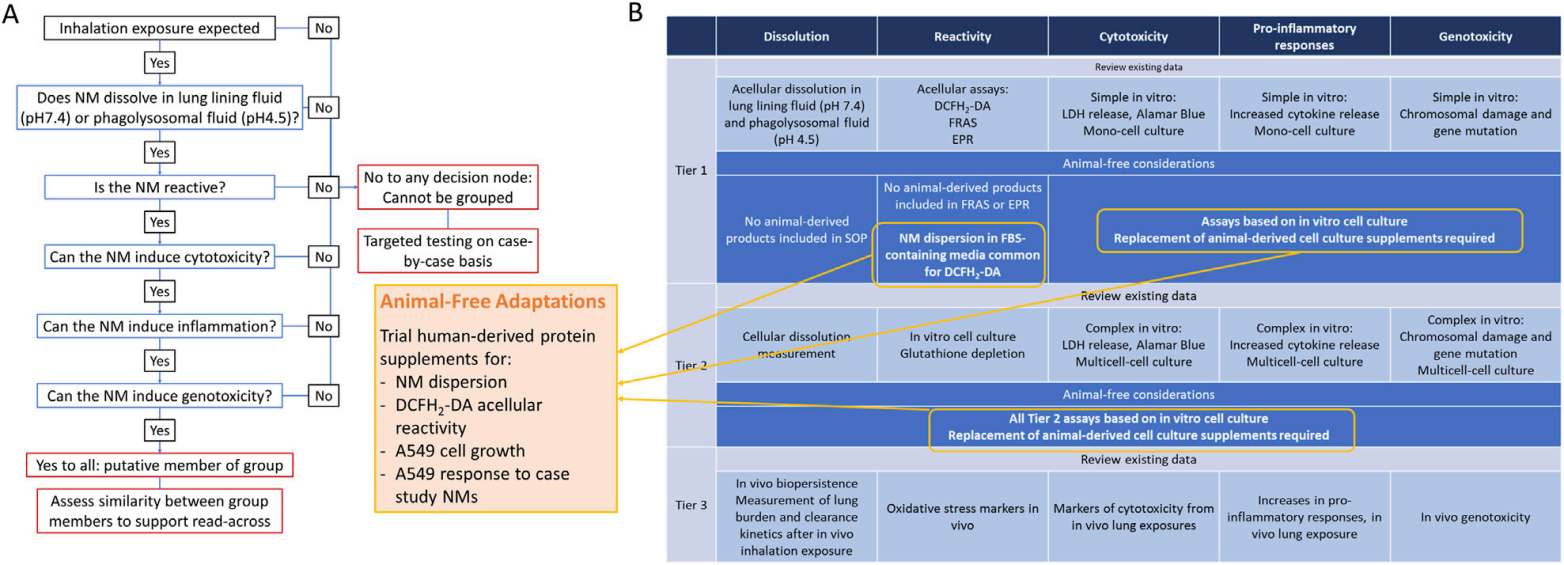
Integrated Approach to Testing and Assessment via inhalation exposure **(A)** and Tiered Testing Strategy **(B)**. Results of audit for use of animal-derived products and suggested adaptations.

The inhalation IATA developed by Braakhuis et al., is supported by a tiered testing strategy to guide the collection of data to group NMs which pose a similar inhalation hazard (Braakhuis et al., 2021). Key characteristics driving toxicity via the inhalation route of exposure were identified including dissolution, reactivity, cytotoxicity, inflammogenicity and genotoxicity. The inclusion of animal-derived products in the methods and models included at each tier of the IATA testing strategy were reviewed and animal-free alternatives were considered (Figure 1). Briefly, protocols recommended for assessment of dissolution do not require animal-derived products and are not considered further (Koltermann-Jülly et al., 2018). A number of assays can be selected for the assessment of intrinsic NM reactivity including electroparamagnetic resonance (EPR), ferric reduction ability of serum (FRAS) assay and the reactive oxygen species (ROS) sensitive probe, DCFH_2_-DA. EPR and FRAS do not require animal-product supplementation. Although not essential for the DCFH_2_-DA assay, NM particle suspensions are routinely prepared in a protein-rich solution to generate a more homogenous dispersion and to replicate *in vitro* cell culture conditions. Bovine serum albumin (BSA) or foetal bovine serum (FBS) are common sources of protein used for this purpose (Boyles et al., 2022). Tier 1 cytotoxicity, inflammogenicity and genotoxicity studies involve the measurement of cell viability, pro-inflammatory cytokine release and chromosomal damage after acute exposure of NMs to a monoculture of a relevant cell line, such as A549 alveolar epithelial cells. Routine maintenance of cells in culture requires protein-rich media to support viability and growth of cells. FBS is the most frequently used protein supplement in cell culture (Jochems et al., 2002).

Based on the audit of the tiered testing strategy the following adaptations were prioritised for further assessment:

1. Preparation of well-dispersed NM suspensions in human platelet lysate (HPL) as an alternative to FBS.
2. Comparison between reactive oxygen species (ROS) production measured by DCFH_2_-DA assay from NMs prepared in HPL *versus* FBS-supplemented media.
3. Evaluation of FRAS assay as an appropriate measure of intrinsic reactivity of NMs compared to DCFH_2_-DA.
4. Impact of replacing FBS with HPL in A549 cell culture.
5. Impact of replacing FBS with HPL in A549 sensitivity to NM case study panel.

### 3.2 Nanomaterial suspension

Dynamic light scattering (DLS) analysis was performed to characterise hydrodynamic diameter and polydispersity index (PDI) of the case study NM panel dispersed in PBS supplemented with 2% FBS or 2% HPL (Table 1). A significant increase (p < 0.05) in both hydrodynamic size and polydispersity of CuO NMs when dispersed in HPL-containing PBS in comparison to FBS was observed. A significant decrease in BaSO_4_- HPL hydrodynamic diameter was recorded however this did not correspond to a change in PDI. No significant differences were noted for ZnO or CeO_2_ when prepared in FBS or HPL-supplemented media.

**TABLE 1:** Size and agglomeration status of NM panel. Hydrodynamic diameter (nm) and Polydispersity Index (PDI) by dynamic light scattering techniques of BaSO_4_, CeO_2_, CuO and ZnO when suspended in PBS with 2% FBS or HPL after sonication by ultra-sonication water bath. Values represent the mean ± SEM (n = 3 individual replicates per treatment). Statistical analysis was performed using an unpaired 2-tailed T-test; *p < 0.05 vs CuO FBS.

	Hydrodynamic size (nm)	+/− SD	t-test	Polydispersity index	+/− SD	t-test
CuO_FBS	225.08	46.94	* p < 0.05	0.58	0.14	* p < 0.05
CuO_HPL	391.72	23.88		0.91	0.08	
ZnO_FBS	502.44	33.13	ns p = 0.16	0.24	0.03	ns p = 0.406
ZnO_HPL	442.53	44.83		0.26	0.01	
CeO_2__FBS	533.74	103.12	ns p = 0.079	0.42	0.08	ns p = 0.103
CeO_2__HPL	348.33	65.69		0.28	0.06	
BaSO_4__FBS	590.20	83.27	* p < 0.05	0.61	0.06	ns p = 0.421
BaSO_4__HPL	383.82	67.45		0.56	0.07	

### 3.3 Acellular reactivity

**FIGURE 2:**
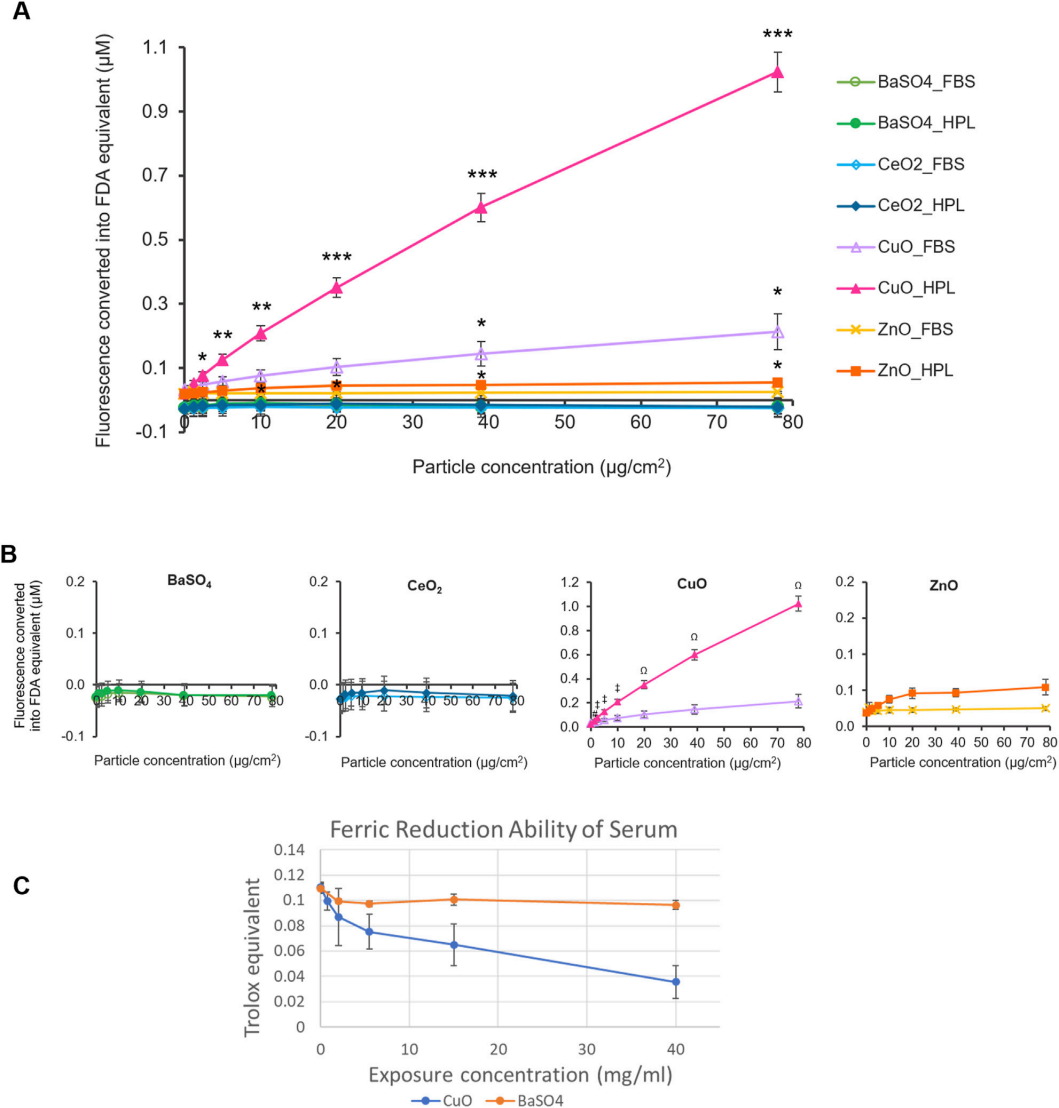
Acellular ROS levels of case study NMs. **(A)** Fluorescence levels of case study NMs, dispersed within 2% FBS- or HPL-supplemented PBS, were detected using the DCFH_2_-DA assay and normalised to standard curves of fluorescein diacetate (FDA) fluorescence. PBS alone was included as the vehicle control. Statistical analysis was performed between each particle concentration and the corresponding vehicle control using an unpaired T-test; *p < 0.05, **p < 0.01 and ***p < 0.001 vs own particle’s vehicle control (0 μg/cm^2^). **(B)** Individual graphs for each case study NM. Data represent the mean ± SEM (n = 3 per treatment). Statistical analysis was performed using a one-way ANOVA test; # (p < 0.05) (#) vs. 1.2 μg/cm^2^ CuO in FBS, p < 0.01 (‡) vs 2.4, 5, and 10 μg/cm^2^ CuO in FBS, p < 0.001 (Ω) vs 20–78 μg/cm^2^ CuO in FBS. **(C)** Antioxidant capacity of serum after incubation with CuO and BaSO_4_ NM expressed as Trolox equivalent ±SEM (n = 3 per treatment).

To assess the reactivity of the case study NMs, the oxidant-sensitive fluorescent probe, DCFH_2_-DA was used to determine the intrinsic ROS production of each case study NM prepared with the different media conditions (Figure 2A). The fluorescent values for each treatment were normalised against the fluorescent signal produced by a standard curve of fluorescein diacetate (FDA).

CuO and ZnO NMs suspended in HPL-containing PBS showed significantly higher fluorescence levels at concentrations of 2.4 μg/cm^2^ and 10 μg/cm^2^ and above, compared to the HPL-PBS no particle control, respectively. Fluorescence levels indicative of ROS production by CuO at 39 and 78 μg/cm^2^ when suspended in FBS were also found to be significantly higher than the FBS vehicle control (p < 0.05). No significant difference was found in any of the BaSO_4_, CeO_2_ samples in either condition or the ZnO samples prepared in FBS-PBS. From this, we identified CuO, in both HPL-PBS and FBS-PBS, and ZnO, in HPL-PBS only, to be ROS-producing NMs. We found that CuO samples prepared in HPL-PBS displayed significantly higher fluorescence levels at particle concentrations from 1.2 μg/cm^2^ and above in comparison to CuO samples dispersed in FBS-PBS. No significant difference was found between any of the other particles when comparing between the means of each particle concentration in FBS or HPL.

Based on comparison of the top concentration of each particle and the vehicle control, we proposed an acellular hazard ranking order for the NMs in their respective media condition as follows: CuO_HPL >> CuO_FBS = ZnO_HPL >> ZnO_FBS > CeO_2__HPL = BaSO_4__HPL = BaSO_4__ FBS = CeO_2__FBS.

Ferric Reduction Ability of Serum (FRAS) assay measures the reduction in antioxidant capacity of human serum after incubation with NMs as an indirect indicator of ROS generation and does not require animal-derived reagents. Similar to the results from DCFH_2_-DA assay, CuO NM caused a reduction of antioxidant capacity, indicating exposure concentration dependent generation of ROS whereas BaSO_4_ did not cause a detectable reduction in antioxidant capacity (Figure 2C). Due to limited available material and the high exposure concentrations required to measure FRAS in NM suspensions the assay was not conducted for other members of the NM panel.

### 3.4 Tier 1 *in vitro* hazard assessment

The impact of switching from animal to human-derived proteins on NM toxicity testing was evaluated using A549 epithelial cells. A549 cells are a human epithelial cell line derived from basal cell carcinoma tissue and are commonly used as a model of alveolar type II epithelial cells (ATII) in respiratory research and toxicity testing of NMs (Smith, 1977; Don Porto Carero et al., 2001).

#### 3.4.1 Impact of HPL on A549 growth rate

**FIGURE 3:**
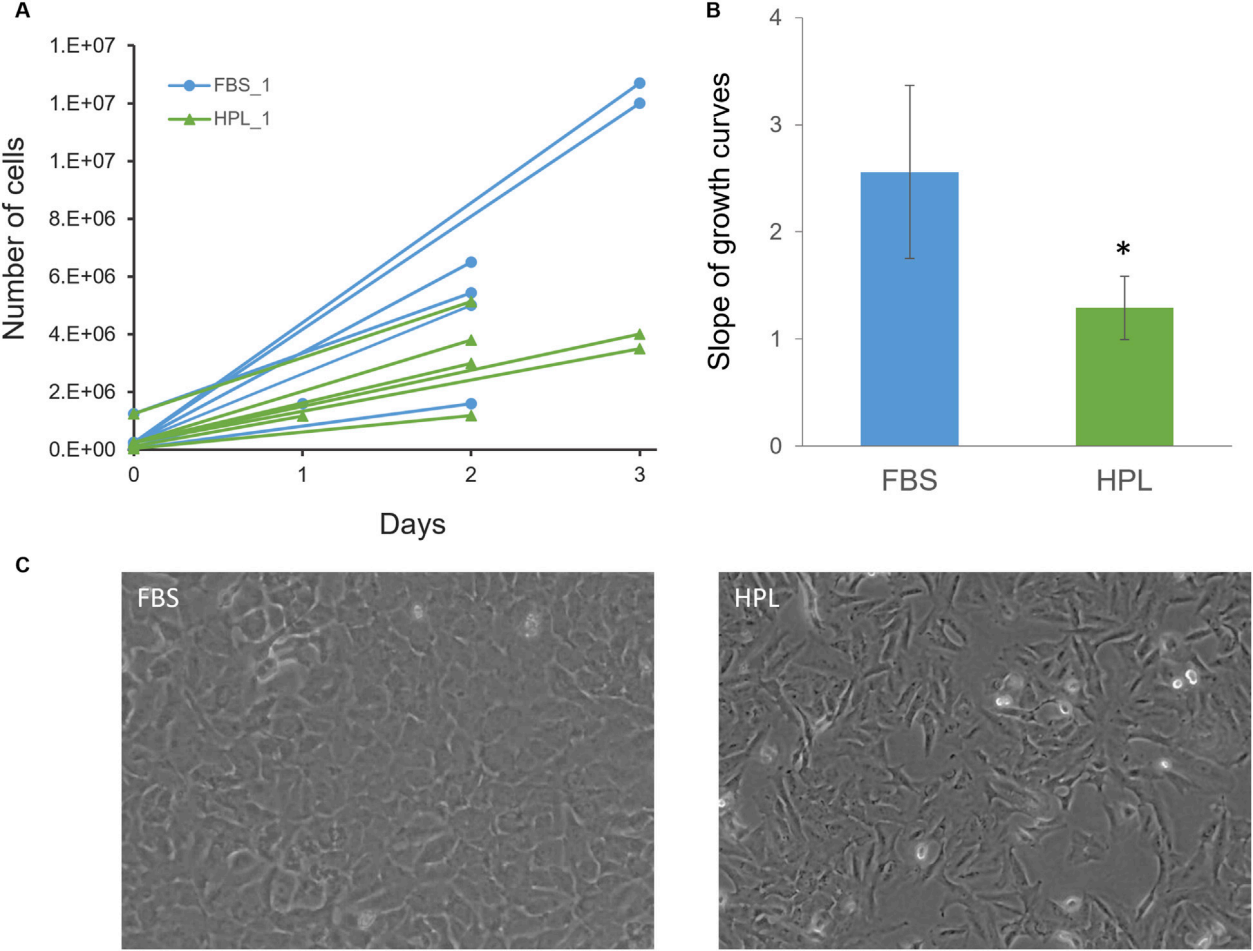
Effect of HPL-supplemented medium on A549 cell growth rates. **(A)** Number of A549 cells grown in medium supplemented with 10% FBS or 5% HPL and counted on days 0, 1, 2 and 3. **(B)** Slope of growth curves. Bars represent mean ± SEM for the slopes of the growth curves of each media condition (n = 7), normalised to cell count on day 0. Statistical analysis was performed using an unpaired T-test; *p < 0.05. **(C)** Light microscopy images of A549 cells which had been cultured in FBS- or HPL-supplemented media.

The effect of switching from media supplementation from 10% FBS to 5% HPL on A549 cells under basal culture conditions was first evaluated. A significantly slower rate of A549 cell growth was observed when cultured in HPL-media (Figures 3A, B). Light microscopy imaging of A549 cells in the two media conditions (Figure 3C) highlighted distinct morphological differences between the two media conditions. Cells grown in FBS supplemented media displayed typical epithelial ‘cobblestone’ morphology while the change to HPL media resulted in cells with a spindle-shaped morphology more commonly observed in mesenchymal cell lines.

#### 3.4.2 Cytoskeletal protein expression

**FIGURE 4:**
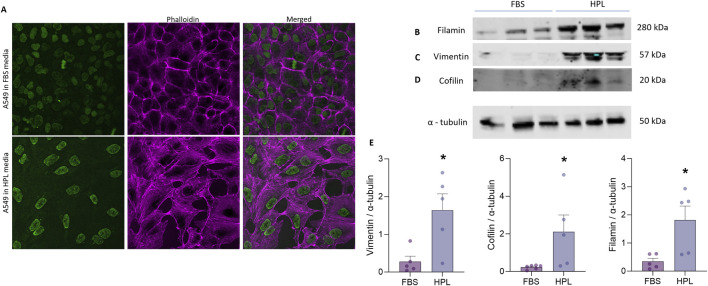
HPL-mediated changes in cytoskeletal protein expression. **(A)** F-Actin expression was qualitatively visualized by immunofluorescent phallodian staining. **(B–D)** Western blot analysis of cytoskeletal protein expression from A549 cell lysates, cultured in FBS or HPL media, was detected anti-vimentin, anti-cofilin, anti-filamin and anti-αSMA antibodies and anti-tubulin as the internal control. Lysates from 3 independent biological replicates were run together on the same gel. Protein bands were visualised using LI-COR Gel Imaging System. **(E)** Relative band intensity of protein expression was normalised compared to loading control and quantified using ImageJ. Data represents the mean +SEM (n = 3). Statistical analysis was performed using an unpaired T-test; *p < 0.01.

To investigate whether changes in the underlying A549 cytoskeleton reflected the observed morphological differences between cells grown under each media condition immunofluorescent staining for filamentous (F)-actin stress fibres was conducted. A qualitative increase in F-actin stress fibre formation in HPL-supplemented media was observed in the spindle-like A549 cells as well as a flattened, larger nuclear morphology (Figure 4A). Western blot analysis of the intermediate filament protein vimentin was significantly increased in cells grown in HPL-supplemented media (Figure 4B). Vimentin is primarily expressed in cells with a mesenchymal phenotype (Ridge et al., 2022). Additional changes in expression levels of key regulators of actin polymerisation were also observed. A significant increase in the actin-binding protein, cofilin was detected in HPL media (Figure 4C). Filamin A which plays a crucial role in organisation and maintenance of cytoskeleton (Sutherland-Smith, 2011) was also significantly increased (Figure 4D). Interestingly an apparent decrease in alpha smooth muscle (αSMA) commonly used as a marker of cells undergoing EMT and adopting a mesenchymal phenotype (Scanlon et al., 2013) was observed in A549 cells grown in HPL-media (Figure 4E).

#### 3.4.3 Wound healing assay

Increased expression in vimentin intermediate filament protein and changes in actin organization suggests the A549 cells are adopting a more motile phenotype which was examined by the wound healing scratch assay. Contrary to expectations the scratch wound closed more quickly in A549 cells grown in FBS compared to A549 cells grown in HPL (Supplementary Figure S1). However, differences in the proliferation rate between cell grown in each media condition (Figure 3) may be a confounding factor obfuscating potentially more subtle differences in motility.

#### 3.4.4 A549 response to nanomaterial exposure

**FIGURE 5:**
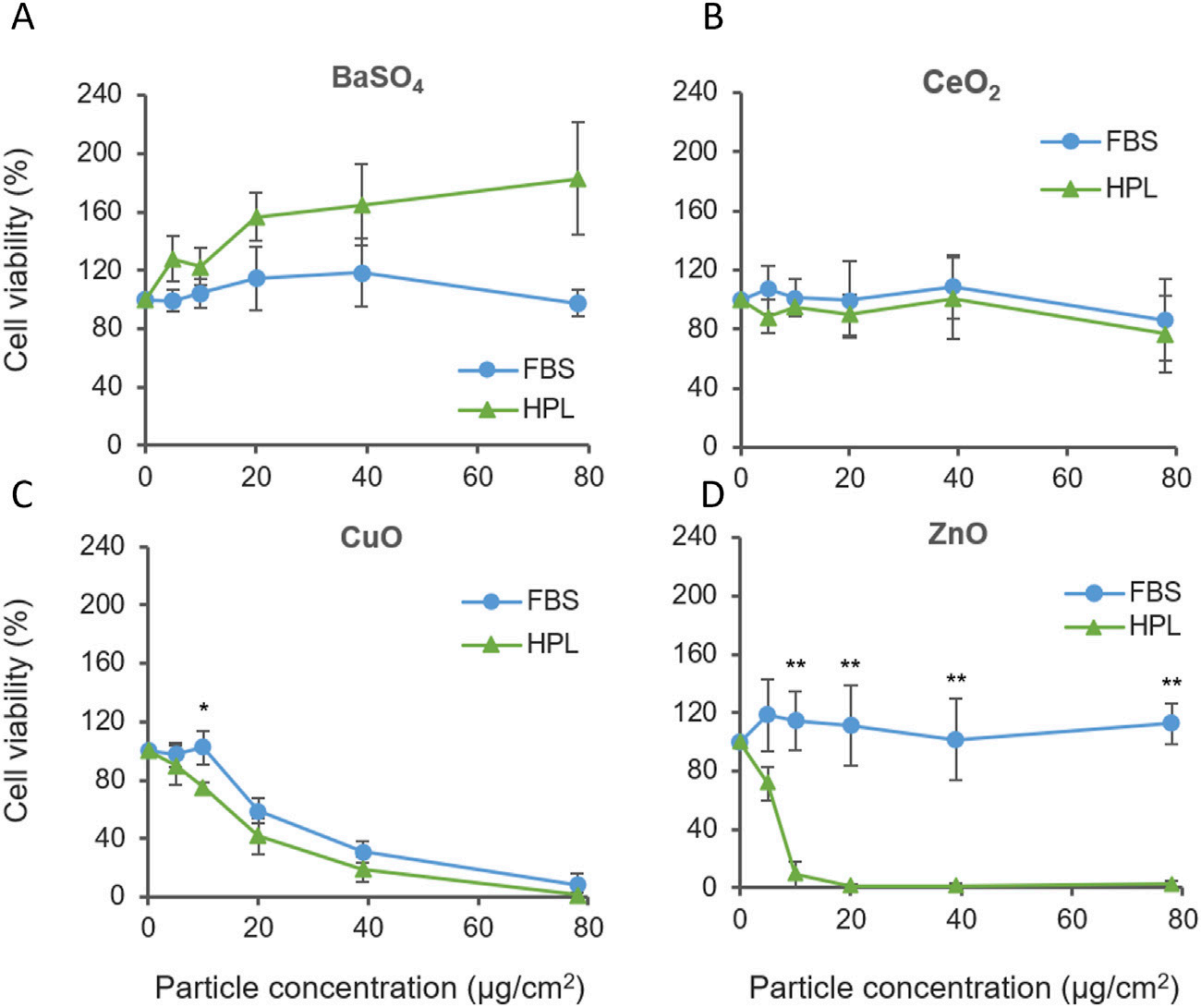
Cell viability of A549 cells cultured in FBS or HPL-media exposed to case study panel of NMs for 24 h **(A)** BaSO4 NM, **(B)** CeO2 NM, **(C)** CuO NM, **(D)** ZnO NM. n = 5 *p < 0.05, **p < 0.01.

A549 cultured in HPL and FBS media were exposed to the case study panel of NMs: ZnO, CuO, CeO_2_ and BaSO_4_. Each NM suspension was prepared in matched media prior to A549 exposure. Cell viability was measured after 24 h exposure (1.2–78 μg/cm^2^ dose range). CuO NMs caused significant levels of cellular cytotoxicity compared to untreated cells from 20 μg/cm^2^, however, no difference was observed between media conditions. Interestingly, increased sensitivity to ZnO NMs was observed when cells were cultured in HPL media (Figure 5A), with significant differences between FBS- and HPL-media conditions observed from 10 μg/cm^2^ to the top concentration of 78 μg/cm^2^ (p < 0.01).

ZnO NM cytotoxicity was also examined over a 5 h treatment period. Elevated levels of cell cytotoxicity were observed within both media conditions however, a dose-dependent reduction in viability was observed only in HPL but not FBS at 5 h. Corresponding brightfield microscopy images show the progressive detachment of the A549 cell monolayer with increasing exposure concentrations of ZnO NM under HPL-media conditions. (Supplementary Figure S2).

Comparison of the relative cytotoxicity of each NMs reported in Figure 5 after incubation with A549 cells grown in FBS and HPL-supplemented media allowed the relative hazard posed by each NM to be ranked. From exposure of A549 cells grown in FBS-supplemented media the following hazard ranking is suggested: CuO >> ZnO > CeO_2_ = BaSO_4_.

Results from A549 cells grown in HPL-supplemented media leads to a different interpretation of NM hazard as suggested by the following hazard ranking: ZnO >> CuO > CeO_2_ > BaSO_4_, directly demonstrating the impact of media supplementation and the conditions under which cells are cultured and exposed to NM can directly impact the outcomes of a hazard assessment.

#### 3.4.5 Particle uptake by FBS and HPL-cultured cells

**FIGURE 6:**
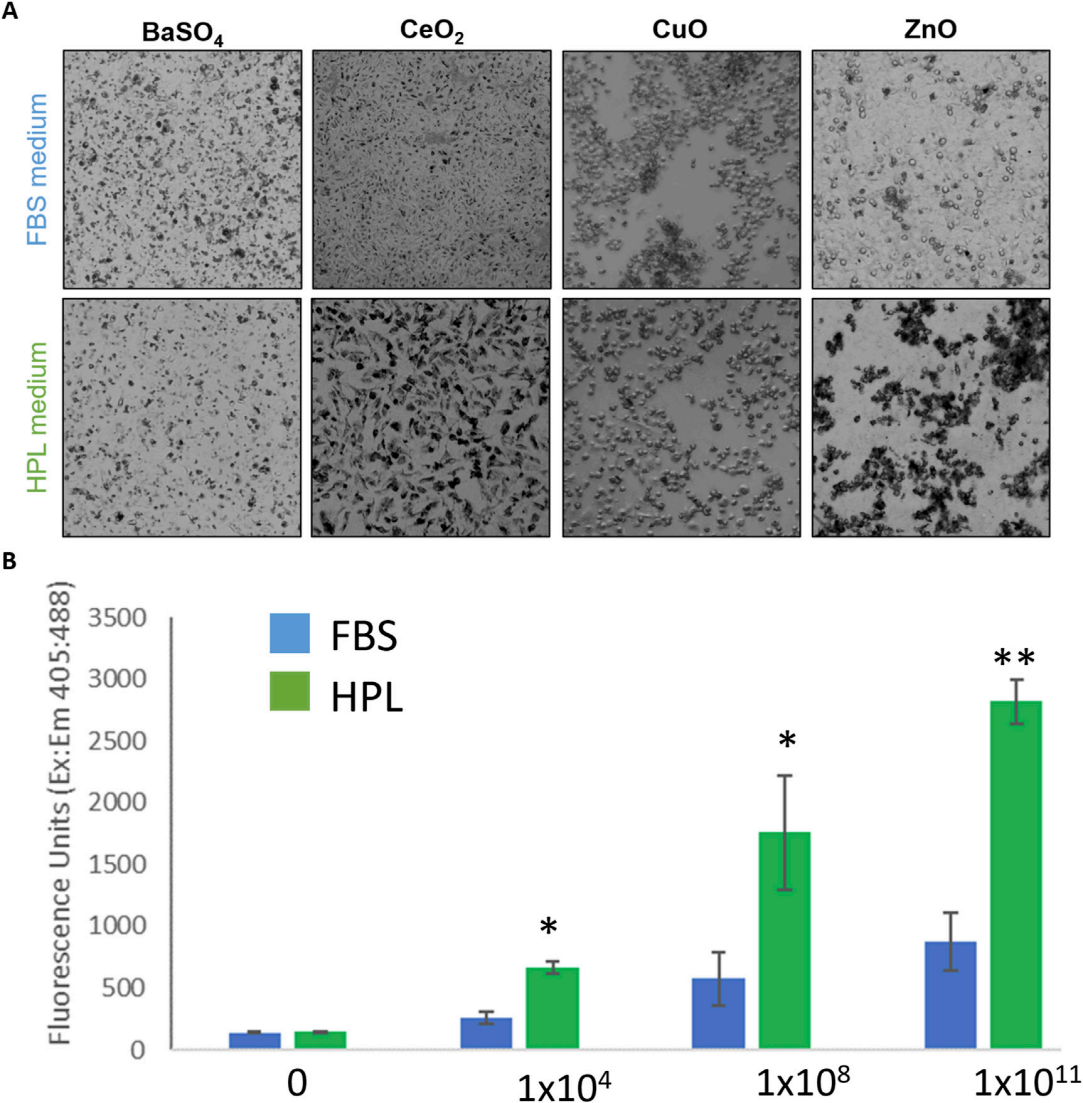
NM uptake by A549 cells in FBS and HPL media. **(A)** Qualitative brightfield microscopy images of FBS and HPL cells exposed to NM panel (10 μg/cm^2^) for 24 h. Differing pattern of uptake observed in cells grown and exposed under each condition. **(B)** Uptake of fluorescent beads measured after 5 h incubation of A549 cells with Fluoresbrite^®^ YG Carboxylate Microspheres 200 nm beads. Statistical analysis was performed using an unpaired t-test compared to FBS *versus* HPL; n = 3 **p < 0.01, *p < 0.05.

After 24-h exposure to the NM panel qualitative differences in particle uptake were observed with an apparently greater level of uptake seen in A549 cells cultured in HPL-media (Figure 6A). It was hypothesized the difference in cytotoxicity which resulted from exposure to ZnO NM under different media conditions may be due to increased particle uptake in HPL-media cells. Uptake of 200 nm fluorescent polystyrene beads was measured using a fluorescent microplate reader after 5 h incubation. Figure 6B shows a significantly greater fluorescent signal in A549 cells cultured in HPL-media compared to FBS-media, which increased with increasing exposure concentration of fluorescent beads.

## 4 Discussion

The aim of this study is to address the pertinent need to reconsider the *status quo* use of animal-derived products in toxicity testing of NMs and to support the timely incorporation of animal-free SOPs into the IATA frameworks and tiered testing strategies which are currently gaining traction and progressing towards validation for regulatory acceptance (Hristozov et al., 2024). The EU complete ban on the use of animal models for the safety assessment of cosmetics since 2013 provided the critical impetus for the rapid development, validation and adoption of alternative models to assess skin irritation and sensitization (Casati et al., 2018). *In vitro* methods that can replace animal testing in the identification of skin sensitisers are now commonly used to generate the required regulatory hazard to bring a product to market. Although considered a success in the promotion of 3Rs principles a recent systematic review of 156 articles utilising *in vitro* methods to assess skin sensitization was carried out by Marigliani et al., who reported the use of several animal-derived products from different species in many protocols, with the use of FBS being cited in 78% of studies (Marigliani et al., 2019a). Therefore, further optimisation of skin sensitization NAMs is still required to completely remove the use of animals from cosmetics testing. Although in their infancy compared to the NAMs used to detect skin sensitization, efforts to develop advanced models to assess hazard of NM and substances via other exposure routes have made significant progress in recent years (Fentem et al., 2021). It is therefore timely to consider the hidden burden of animal use at this stage of NAM development to ensure animal-derived products are excluded where possible. To meet this need we conducted an initial audit of an IATA developed for the assessment of NMs hazard via inhalation and identified a number of key areas where animal reagents such as BSA or FBS are commonly used, from initial preparation of NM suspensions to *in vitro* cell-based assays. Critical assays were then selected for further assessment to explore the feasibility of removing and/or replacing animal-derived components, focussing on the replacement of FBS with HPL.

Worldwide production of FBS is estimated at approximately 600,000 to 800,000 L collected from around 1 to 2 million foetuses each year (Jochems et al., 2002). Although included in *status quo* cell culture protocols FBS is a complex mixture of different factors and introduces a degree of complexity and variability that can have a substantial impact on experimental results. Of note, albumin a major protein constituent of FBS contains high levels of antioxidants and can act as a protective buffer to cellular stresses and influence the detection of particle-mediated oxidative stress (Boyles et al., 2022). Reducing the potential for xenobiotic immunological responses is also particularly relevant for NM toxicity testing to ensure potential hazard is accurately identified and to mitigate against artefactual responses.

Issues with data reproducibility have been shown to arise from batch-to-batch variation between FBS sources leading to inconsistencies in results (Barosova et al., 2021). As a result, the transition to FBS-free media is encouraged by scientific organizations worldwide; the Organisation for Economic Co-operation and Development (OECD) began to discourage the use of FBS in 2017, especially for human health risk assessments of chemicals (Pedersen and Fant, 2018). Additionally, to address both reproducibility and ethical concerns related to the use of FBS the EU Reference Laboratory for Alternatives to Animal Testing (EURL ECVAM) Scientific Advisory Committee (ESAC) requires justification when non-animal alternatives to serum are not used in methods forwarded to the organization for validation/pre-validation (Directive, 2010, 2022; ECVAM News and Views, 2008). Numerous options are now available to act as replacements for FBS and animal-derived components in cell culture including an increasing number of commercially available media formulations of chemically defined, animal component-free, or xeno-free media and supplements. The Fetal Calf Serum-Free Database lists commercially available serum-free media and summarises medium compositions from the scientific literature for various cell types (Utrecht, 2024). For our study we opted for HPL as a cost-effective replacement for FBS, however acknowledge the issue of batch-to-batch variation remains a concern.

The essential physiological role of platelets in wound healing and tissue repair underlies the rationale for the use of human platelet derivatives in cell culture (Burnouf et al., 2016). HPL has emerged as a promising alternative to FBS for cell culture applications, offering advantages in safety, efficacy, and ethical considerations (Ben Azouna et al., 2012; Brachtl et al., 2022; Viau et al., 2017; Liau et al., 2021; de Wildt et al., 2022). Studies have demonstrated that HPL is rich in growth factors that promote cell proliferation, making it a viable substitute for FBS as a culture medium (37-39) title = supplement_ (Murphy et al., 2023; Guiotto et al., 2020; Bieback et al., 2009) > supplement (Murphy et al., 2023; Guiotto et al., 2020; Bieback et al., 2009) however, as an equally complex protein mixture but with a differing composition profile, the impact of replacing FBS with HPL needs to be well understood to support a change in protocol and to ensure fidelity of hazard results obtained.

Here we observed a significant increase in both the hydrodynamic size and polydispersity of CuO particles in HPL compared to CuO in FBS, suggesting a difference in NM agglomeration. NMs have the ability to adsorb proteins and other biomolecules from biological media on to their surface, forming a ‘protein corona’ which dictates particle-particle and particle-cell interactions (Brachtl et al., 2022; Zhang et al., 2024). Due to the varying composition of biomolecules in different media preparations, different protein coronas can be created (Ge et al., 2015). Furthermore, factors such as the size, surface chemistry (Mortensen et al., 2013), charge and shape of the NM, as well as the binding affinity of particular media proteins for specific NMs, can majorly impact the formation and composition of the protein corona (Ahsan et al., 2018; Kopac, 2021) which may explain the interesting observation that switching to HPL lead to a measurable increase in hydrodynamic diameter and polydispersity of CuO NM but decrease of BaSO_4_ diameter. Once formed, the protein corona can influence various characteristics of the NM such as the agglomeration of particles, e.g., in a study from Vertegel et al., (Vertegel et al., 2004), lysozymes were shown to bind to silica NMs and cause enhanced NM aggregation, with Park et al. (Park, 2020), postulating that this was due to the interaction of positively charged residues on the bound lysozymes interacting with other proteins. Adsorbed proteins are also able to act as physical bridges between NMs and thus promote agglomeration (Falahati et al., 2019) while on the other hand serum albumin has been shown to coat some NMs and decrease agglomeration through inhibition of electrostatic and attractive forces between individual particles (Zook et al., 2011). Particle agglomeration is a key factor that must be considered in nanotoxicity testing, as it has been shown to affect NP toxicity and reactivity. Murugadoss et al. (Murugadoss et al., 2020), reported a size-dependent decrease in glutathione levels and increase in IL-8 and IL-1β levels and increase in DNA damage when THP-1 macrophages were exposed to large and smaller TiO_2_ agglomerates. Furthermore, TiO_2_ prepared in various media reportedly caused differences in particle agglomeration, cellular interactions and chromosomal damage in BEAS-2B cells, with differences attributable to changes in protein corona (Prasad et al., 2013).

Evaluation of intrinsic NM reactivity is a core component of the inhalation IATA and considered a key driver of NM toxicity. Ensuring the methods employed to assess NM reactivity are sufficiently robust to differentiate high from low reactivity NM is therefore critical. Here we showed the choice of media supplement used in the preparation of NMs can result in significantly different responses when ROS production is detected by the DCFH_2_-DA probe which will fundamentally affect the interpretation of relative hazard or NM hazard ranking. Therefore, care should be taken when comparing DCFH_2_-DA results from across the literature. We also demonstrated the FRAS assay, which does not require animal-derived products, can differentiate between high reactivity CuO and low reactivity BaSO_4_ confirming results previously reported by (Ag Seleci et al., 2022). Interesting in this study the authors reported significant reactivity of ZnO when measured by the FRAS assay but not when the DCFH_2_-DA assay was used, suggesting the FRAS assay is a more sensitive read-out. It is tempting to recommend the FRAS assay as the first-choice reactivity assay in ‘humanised’ IATA however a number of key limitations should first be considered. The FRAS assay may not be suitable for all material types, e.g., colorimetric assays, including the FRAS assay, are not recommended for NM with high absorption coefficient (i.e., where traces of NM may produce a false positive result due to optical interference) as demonstrated with NM pigments in (Ag Seleci et al., 2022). Another challenge associated with FRAS assay are comparatively high exposure doses required to obtain a signal (e.g., 60 mg per 1.5 mL of serum as the highest dose) which could pose a problem when only a limited amount of the material is available.

We focussed our study on the IATA tailored towards inhalation, as the primary route of particle exposure. The IATA recommends a tiered approach to toxicity testing initially utilising simple monocell cultures of a cell-line relevant to the exposure route to screen acute toxicity and pro-inflammatory and/or genotox changes. The A549 cell-line isolated from a basal cell adenocarcinoma from a 58-year old Caucasian male (Lieber et al., 1976) are commonly used as a model system for Type II alveolar epithelial cells, however A549 cell phenotype and behaviour has been shown to be manipulated by culture conditions (Cooper et al., 2016; Wu et al., 2017) further exemplified by the reduction of cell growth rate and change of morphology we have observed here.

It has been reported that HPL in general has the higher concentration of growth factors than any other cell culture supplements including FBS (Guiotto et al., 2020). Correspondingly, the majority of recent reports assessing the use of HPL in expansion of mesenchymal stem cells agree that HPL supports cell expansion to a higher degree, compared to FBS, cells proliferate faster and maintain undifferentiated phenotype (Guiotto et al., 2020). Hildner et al. (2015) reported both human articular chondrocytes and human adipose-derived stem cells cultured with 5% HPL showed strongly enhanced proliferation rates compared to cells grown in 10% FBS. Rauch et al. (2011), showed almost identical growth promoting effect of 5% HPL is compared to that of 10% FBS for renal epithelial cells and human leukemia cell lines. Hofbauer et al. (2014), demonstrated use of HPL on different EC types did not reveal any substantial negative effects on EC growth however a slight decrease in metabolic activity was noted. Conversely here we see a reduction in A549 cell growth rate and change in morphology. To normalise our comparative cell culture conditions we opted to reduce the concentration of HPL to more closely align with the total protein content of FBS which may have led to the reduction in proliferation rate, however this adjustment was based on total protein content and not tailored to matching levels of specific proteins with known functional impact on cell growth. Comprehensive characterisation of FBS and HPL-supplemented media composition should be conducted in future mechanistic studies to elucidate the impact of specific factors present in each media. It has also been reported that HPL may stimulate cells to mature which may account for reduction in proliferation as cells differentiate (Bieback et al., 2009). The evident change in morphology and expression of structural proteins identified in our study may therefore be indicative of differentiation or phenotypic switching to a less proliferative state. Confirming the relative reduction of cell proliferation observed here, Fazzina et al. (2016), showed that FBS resulted in a faster replication rate for haematopoietic cell lines (KG-1, K562, JURKAT, HL-60), with an average of doubling time 14% higher than HPL cultures. A lack of standardisation in preparation of HPL and protocols for use are likely to be responsible for the contradictory impacts on cell culture proliferation and differentiation reported in the literature. Here we report the impact of different media composition on cell growth, phenotype and behaviour using a direct approach to switching from FBS to HPL-supplemented media. It would be interesting to compare the impact of different HPL concentrations and alternative strategies for adaption periods, e.g., gradual, or staggered replacement over prolonged time (Marigliani et al., 2019b; Weber et al., 2022). Suggesting an optimal protocol for adaptation to HPL-supplemented media was considered out with the scope of this manuscript rather the results reported here provide direct evidence for the need to careful consideration of cell culture conditions and processes when developing NAMs which deviate from standardised protocols such as the growth of cells in FBS-containing media.

Commercially available HPL usually consists of a pool of at least three different blood donors, to get a standardised product and prevent undesired donor-dependent characteristics. Hesler et al. (2019), tested effect of non-pooled, single patient-derived HPL on A549, HepG2 and Caco-2 human cell lines. The relative proliferation of A549 cells decreased when cultured in the presence of 10% single doner HPL (74.5 + 10.1%), 6% single doner HPL (74.0 + 11.0%) and 10% human serum (78.7 + 13.4%) compared to cells grown in 10% FBS. Here we compared a single batch of FBS with a single batch of HPL as a limited, proof-of-concept to highlight the need to consideration of media composition in the interpretation of hazard results. Future detailed studies considering impact of batch-to-batch variation on the phenotypic changes reported here will be required to delineate the critical factors driving different responses, for example, additional mechanistic studies tailored to better interpret the influence of the protein corona derived from different serum sources on nanomaterial toxicity.

Discrepancies between studies highlight the fundamental limitation of all protein mixtures used as cell culture supplements regardless of origin. Hormones, growth factors and other signalling molecules are abundant in serum, but tightly regulated in interstitial fluid in which cells in the body are bathed (Baker, 2016). The microenvironment of cells, most of which are not directly exposed to serum and its higher concentration of proteins *in vivo*, may be better modelled in serum-free cultures compared to those supplemented with FBS (Muoio et al., 2021). However, the growth and maintenance of cell lines using serum-free media is not always possible, or pragmatic. Most serum-free formulations apply only to a specific cell type or closely related group of cell lines, may be prohibitively expensive for widespread application in cell culture lab where multiple cell lines and types are used routinely. As novel media formulations which may have broader applications across a wide variety of cells start to become available (Rafnsdóttir et al., 2023; Weber et al., 2024) it would be interesting to assess the comparative growth, phenotype and behaviour of cells and impact of use of new formulations on NAM performance. However, weaning cells off serum and transferring to novel media formulations can take time, resources, and effort. Chary et al. (2022) compared a number of commercial serum-free defined media and showed that A549 cells can be successfully transitioned to FBS-free media under submerged and air-liquid interface conditions, however only 2 out of 4 media tested were effective. Each serum-free formulation resulted in distinct cell phenotypes typified by differences in doubling time ranging from 21.3 ± 1.5 h (DMEM +10% FBS) to 51 ± 6.6 h (CnTPrime Airway). A change in morphology was also observed with A549 cells grown in either DMEM +10% FBS or X-VIVO™ media showing a similar morphology of smaller, cuboidal cells (about 10–20 μm in diameter) whereas CnT-Prime Airway cultured cells were a heterogeneous mixture of large cells (>50 μm in diameter), medium cells (about 20–50 μm in diameter), and smaller, cuboidal cells. Significant differences in cell sensitivity to toxic insult were also reported as dependent on culture conditions. The authors concluded that A549 cells cultured in CnTPrime Airway may lose their adenocarcinomic phenotype in favour of differentiation towards an ATI and ATII epithelial cell-like phenotype. Therefore, the choice of media and culture conditions may have an impact on the physiological relevance of a NAM.

To support the replacement of FBS in cell culture systems used in toxicity testing it is critical to assess how changes in cell phenotype or functionality stimulated by the new culture environment may affect the response of cells to toxic agents and therefore alter the perception of hazard compared to other studies or previous data collected. To address this important factor, we assessed the cytotoxicity of a panel of well characterised NMs prepared as suspensions in FBS- or HPL-supplemented media and exposed to A549 cells cultured under each media condition. Interestingly although CuO NM showed much greater ROS production in the DCFH_2_-DA assay when prepared in HPL-medium compared to FBS this was not replicated in a corresponding increase in cytotoxicity suggesting the level of ROS production under FBS-media conditions is sufficient to trigger mechanisms of cell death within the parameters of the experimental set-up (NM exposure concentration x exposure time). Therefore, although the acellular reactivity assay ranks CuO-HPL higher in potential hazard the toxicology implications are comparable to CuO-FBS in a cellular system. On the other hand, ZnO-HPL and ZnO-FBS both produced very low levels of acellular ROS, however ZnO-HPL was significantly more toxic to A549 cells grown in HPL media compared to the cytotoxicity caused by ZnO-FBS exposure to A549 cells cultures in media with FBS. The degree of cytotoxicity observed resulted in a necessary re-ordering of the hazard ranking of the NM panel when potential to cause cytotoxicity to A549 cells was compared; CuO NM, the highest toxicity material identified under FBS conditions, was surpassed by ZnO when FBS was switched for HPL. Furthermore, the hazard ranking for all NMs generated from cytotoxicity study was different when compared to acellular DCFH_2_-DA reactivity (DCFH_2_-DA: CuO_HPL >> CuO_FBS = ZnO_HPL >> ZnO_FBS > CeO_2__HPL = BaSO_4__HPL = BaSO_4__ FBS = CeO_2__FBS *versus* cytotoxicity: CuO >> ZnO > CeO_2_ = BaSO_4_ in FBS-media or ZnO >> CuO > CeO_2_ > BaSO_4_ in HPL-media).

The protein corona can impact the NM toxicity profile via altering the nano-bio interface; alternative epitopes on the adsorbed proteins can become exposed upon binding to the NM which can allow for binding to different receptors (Ge et al., 2015) altering uptake of NMs in biological systems, as well as their distribution and clearance. Adsorption of different protein preparations (human serum albumin (HSA), bovine serum albumin (BSA), high-density lipoprotein (HDL) to the surface of silver NPs were shown to induce conformational changes in the structures of bound proteins, for example, the number of α-helices decreased in both HSA and BSA but increased in HDL, whereas the number of β-sheets remained unchanged in all 3 protein coronas. This, in fact, altered the cellular uptake of NPs (specifically through scavenger receptors) and initiated cytotoxicity and an inflammatory response (increased mRNA expression of the pro-inflammatory cytokine IL-6) in *vitro* cellular models (epithelial and endothelial cells) (Shannahan et al., 2015). These studies along with the data presented here exemplify how a change in protein corona due to a change in NM suspension media can influence biological responses at the nano-bio interface should be carefully considered when altering media composition. We hypothesise differences in particle uptake mechanisms triggered by the ZnO-HPL protein corona directly accounts for the increase in cytotoxicity of the ZnO NM compared to FBS-media conditions (Hassanian et al., 2021). It is well acknowledged that ZnO NM mediated toxicity is largely due to the ‘Trojan Horse’ mechanism which describes how the internalisation of ZnO NM leads to rapid dissolution of ZnO NM to highly reactive Zn^2+^ in the low pH of the phagolysosome (Sabella et al., 2014). Therefore, an increase in the rate of ZnO NM uptake can account for the more rapid onset of cell death observed in response to ZnO-HPL compared to ZnO-FBS.

The results of our nanotoxicity testing demonstrate how differences in cell culture set-up can have a fundamental impact on NM hazard assessment with implications for the interpretation of results both within specific assay and across a tiered testing strategy. Therefore, we consider it possible to remove and replace animal derived products from the inhalation IATA while acknowledging that the switch may greatly affect both cell growth a phenotype as well the results of toxicity assays which will need to be carefully considered when interpreting the results of both acellular and cellular toxicity assays in comparison to previously generated hazard data. Further work needs to be done to investigate in detail the impact of HPL as a protein source on cell growth and behaviour and the nano-bio interface. A better understanding of how changes in medium composition may affect the interpretation of specific NM hazard but also importantly *in vitro* assay predictability of human hazard will be fundamental to promote the adoption of NAMs to replace *in vivo* models for understanding risk.

## Data Availability

The datasets presented in this study can be found in online repositories. The names of the repository/repositories and accession number(s) can be found in the article/Supplementary Material.
