# Supplementary material for: Humanising nanotoxicology: replacement of animal-derived products in the application of integrated approaches to testing and assessment of nanomaterial inhalation hazard
Source: Front Bioeng Biotechnol. 2025 Feb 12;13:1526808. doi: 10.3389/fbioe.2025.1526808 (PMC11861073; doi:10.3389/fbioe.2025.1526808)
Supplement: Supplementary file 1 [file DataSheet1.docx]

Supplemental Information


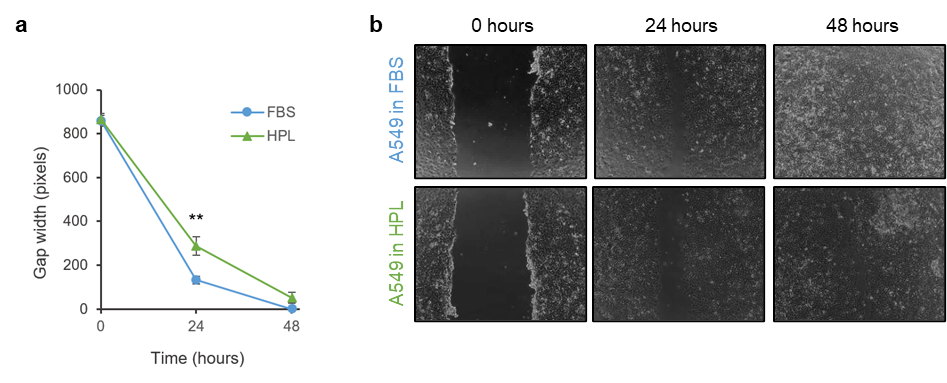


**Fig. S1:** Effect of HPL on A549 cell wound-healing. (a) Quantitative analysis of in vitro scratch wound-healing assay using ImageJ to measure the difference in gap width between media conditions at 0-, 24-, or 48-hr timepoints. Data represents the mean ± SEM (n = 12 independent biological replicates). Statistical analysis was performed using an unpaired T-test; **p < 0.01 vs. 24hr FBS. (b) Light microscopy images corresponding to 0-, 24-, and 48-hr timepoints in the different media conditions.


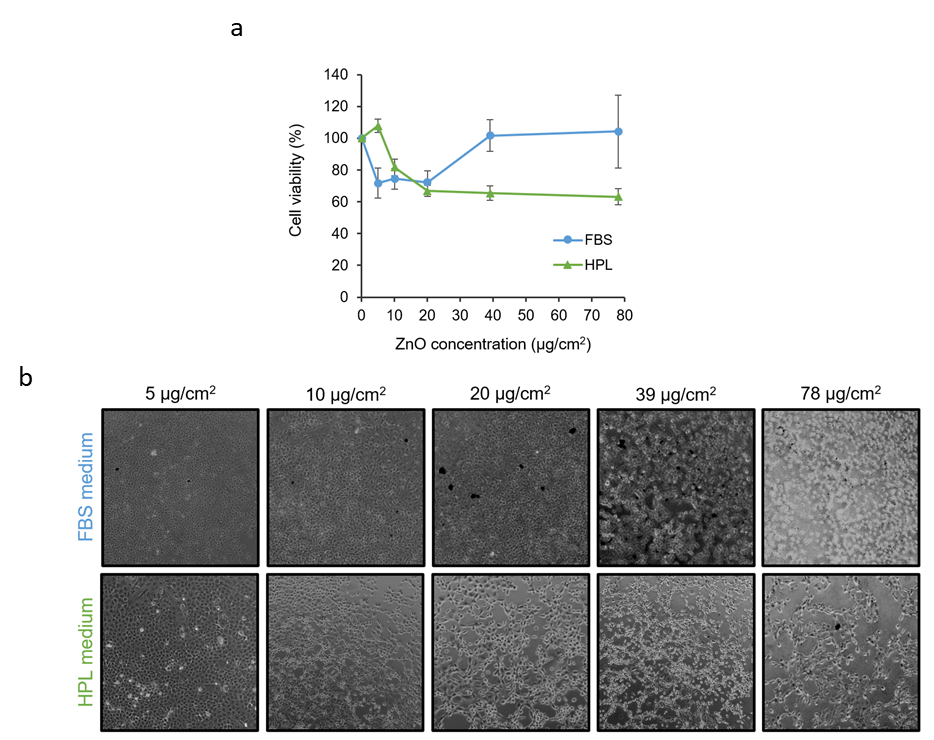


**Fig. S2:** ZnO cytotoxicity after 5 hours exposure (a) Cell viability assay of A549 cells, cultured in FBS or HPL supplemented media, after 5 hours treatment with a range of ZnO concentrations (0 – 78 µg/cm2). (b) Corresponding brightfield microscopy images.
